# Comparative Analysis of Enzyme Production Patterns of Lignocellulose Degradation of Two White Rot Fungi: *Obba rivulosa* and *Gelatoporia subvermispora*

**DOI:** 10.3390/biom12081017

**Published:** 2022-07-22

**Authors:** Mila Marinovíc, Marcos Di Falco, Maria Victoria Aguilar Pontes, András Gorzsás, Adrian Tsang, Ronald P. de Vries, Miia R. Mäkelä, Kristiina Hildén

**Affiliations:** 1 Department of Microbiology, Faculty of Agriculture and Forestry, University of Helsinki, 00790 Helsinki, Finland; mila.marinovic@helsinki.fi (M.M.); miia.r.makela@helsinki.fi (M.R.M.); 2 Centre for Structural and Functional Genomics, Concordia University, Montréal, QC H4B 1R6, Canada; marcos.difalco@concordia.ca (M.D.F.); adrian.tsang@concordia.ca (A.T.); 3 Fungal Physiology, Westerdijk Fungal Biodiversity Institute & Fungal Molecular Physiology, Utrecht University, Uppsalalaan 8, 3584 CT Utrecht, The Netherlands; b52agpom@uco.es (M.V.A.P.); r.devries@wi.knaw.nl (R.P.d.V.); 4 Department of Chemistry, Umeå University, 901 87 Umeå, Sweden; andras.gorzsas@umu.se

**Keywords:** *Obba rivulosa*, *Gelatoporia subvermispora*, proteome, CAZymes, lignin biodegradation, LC-MS/MS, white rot fungi

## Abstract

The unique ability of basidiomycete white rot fungi to degrade all components of plant cell walls makes them indispensable organisms in the global carbon cycle. In this study, we analyzed the proteomes of two closely related white rot fungi, *Obba rivulosa* and *Gelatoporia subvermispora*, during eight-week cultivation on solid spruce wood. Plant cell wall degrading carbohydrate-active enzymes (CAZymes) represented approximately 5% of the total proteins in both species. A core set of orthologous plant cell wall degrading CAZymes was shared between these species on spruce suggesting a conserved plant biomass degradation approach in this clade of basidiomycete fungi. However, differences in time-dependent production of plant cell wall degrading enzymes may be due to differences among initial growth rates of these species on solid spruce wood. The obtained results provide insight into specific enzymes and enzyme sets that are produced during the degradation of solid spruce wood in these fungi. These findings expand the knowledge on enzyme production in nature-mimicking conditions and may contribute to the exploitation of white rot fungi and their enzymes for biotechnological applications.

## 1. Introduction

Wood degradation by fungi is a significant process in terrestrial environments with respect to the cycling of carbon and other nutrients [1,2]. The wood cell walls are composed of the three main polysaccharide components: cellulose, hemicellulose, and pectin, as well as the aromatic polymer lignin. While many fungi can degrade wood cell wall polysaccharides, only basidiomycetous white rot fungi can efficiently degrade lignin [3]. This is due to their unique ability to produce extracellular lignin modifying peroxidases, which catalyze unspecific oxidative reactions to degrade the heterogeneous lignin structures. Moreover, white rot fungal genomes possess several hydrogen peroxide-producing enzymes needed for the activity of the lignin modifying peroxidases, in addition to a wide array of carbohydrate active enzymes (CAZymes) targeted to lignocellulosic polysaccharides (www.cazy.org) that also is produced by a broader range of fungi [4].

Two types of wood decay patterns have been suggested among white rot fungi. In the so-called simultaneous degradation, lignin and (hemi)cellulose are depolymerized at the same time, as exemplified by the action of the well-studied white rot fungus *Phanerochaete chrysosporium* [5]. In contrast, some white rot species, such as *Gelatoporia (Ceriporiopsis) subvermispora* and *Obba rivulosa*, have been suggested to cause selective lignin degradation by preferential removal of lignin before wood polysaccharides [6,7,8]. Selective lignin degradation is typically observed in the early stages of fungal growth, but physical and chemical conditions of the substrate, such as temperature, moisture, and nitrogen and oxygen content, also affect the selectivity of lignin degradation [9,10]. While definitive enzymatic mechanisms behind the selective degradation remain uncertain, increased oxidoreductase and diminished cellulolytic potential have been suggested to differentiate selective delignifiers from species that simultaneously degrade all lignocellulose polymers [11].

*Gelatoporia subvermispora* and *O. rivulosa* are closely related species from the Gelatoporia clade, which is an evolutionarily distinctive group within the order Polyporales, comprising a small group of white rot wood-inhabiting fungi [12,13]. Genome content in terms of putative plant cell wall degrading enzyme (PCWDE) encoding genes suggests that *G. subvermispora* and *O. rivulosa* have similar potential for lignocellulose decay [11,14,15].

Orthologs are homologous genes or proteins that are shared among related species. They are derived from a common ancestral gene that has diverged after the speciation event but with retention of similar biological function [16]. Ortholog analysis is a useful tool in comparative proteomic studies because it can highlight genes shared among species that are important for conserved biological processes. It also can reveal which genes are unique for a particular subset of fungi, such as fungi with a specific lifestyle. As an example, closely related ascomycete species from the genus *Aspergillus* with highly similar genomic potential have been shown to markedly differ in their enzymatic approaches for plant biomass degradation by producing nonorthologous CAZymes [17].

In this study, we wanted to test whether this also is true for closely related wood degrading white rot fungi. For this, we explored the proteomes of the genome-sequenced monokaryotic strains of *G. subvermispora* RP-B and *O. rivulosa* 3A-2 [11,14] during an 8-week cultivation on solid spruce wood sticks, thus mimicking the environmental conditions these fungi encounter in their natural habitat. Temporal production of plant cell wall targeted CAZymes was detected by LC-MS/MS and a comparative analysis of CAZyme orthologs in *O. rivulosa* and *G. subvermispora* was performed to evaluate how similar enzymatic machineries these related white rot fungi employ for spruce wood degradation.

## 2. Materials and Methods

### 2.1. Fungal Strains and Cultivation Conditions

*Obba rivulosa* monokaryon 3A-2 (FBCC1032), derived from the dikaryotic *O. rivulosa* strain T241i (FBCC939), was obtained from the HAMBI Fungal Biotechnology Culture Collection, University of Helsinki, Finland (fbcc@helsinki.fi). *Gelatoporia* (*Ceriporiopsis*) *subvermispora* monokaryon FP-105752 (RP-B), derived from the parental dikaryotic strain 105752 [18], was a kind gift from Dr. Dan Cullen, Forest Products Laboratory, Madison, WI, USA.

The fungi were maintained on 2% malt extract agar plates (MEA; 2% (*w*/*v*) malt extract, 2% (*w*/*v*) agar). Pre-cultivations contained 100 mL low nitrogen–asparagine–succinate (LN-AS) medium, pH 4.5 [19], supplemented with 0.05% glycerol in 250 mL Erlenmeyer flasks, and inoculated with five mycelium-covered agar plugs (Ø 7 mm) for 7 days at 28 °C. Pre-cultivations were homogenized (Waring Blender, Torrington, CT, USA) [20] and solid spruce wood cultivations containing 2 g (dry weight) of Norway spruce (*Picea abies*) sapwood wood sticks (2 × 0.2 × 0.3 cm) on 1% (*w*/*v*) water agar in Erlenmeyer flasks were inoculated with 4 mL of mycelial suspension (Supplementary Figure S1). Moisture content of the cultivation was adjusted to 60%. Cultivations were performed as duplicates and incubated in dark for 2, 4, and 8 weeks at 28 °C. 

### 2.2. Protein Extraction and LC-MS/MS Identification

For proteome analysis, duplicate fungal spruce wood cultures incubated for 2, 4, and 8 weeks were ground (A11 Basic analytical mill, IKA, Merck, Darmstadt, Germany) under liquid nitrogen. The milled cultures were extracted with 40 mL 25 mM potassium phosphate buffer, pH 7.0, under agitation (150 rpm) at 4 °C for 2 h. The extract was filtered (GF/C glass fiber filters, Whatman) and proteins in the liquid fraction were precipitated with 20% trichloroacetic acid (Sigma-Aldrich, St. Louis, MI, USA), 20 mM dithiothreitol (DTT, Sigma Aldrich), and 80% acetone (Sigma-Aldrich) solution, resuspended and reprecipitated with 20 mM DTT and 80% acetone solution as described previously [21].

Ten micrograms of protein were resuspended in 0.25% Anionic Acid Labile Surfactant 26 I (AALS I, Progenta^TM^, Protea Biosciences, Inc., Morgantown, WV, USA) detergent prepared in 200 mM ammonium bicarbonate buffer, pH 7.8, loaded and separated on 12% SDS-PAGE gels for subsequent in-gel trypsin digestion as previously described [22]. Protein separation was stopped as soon as the 250 kDa marker could be seen entering the separating gel. The complete lane, delimited by the bromophenol blue front at the bottom and the start of the separating gel at the top (about 1.5 cm), was cut and processed for in-gel digestion as a whole.

In-gel digested peptide extracts from two biological replicate cultures were analyzed on an LTQ-Velos-Orbitrap mass spectrometer (Thermo-Fisher, San Jose, CA, USA), and the acquired MS/MS data were searched against databases at JGI Mycocosm for the *O. rivulosa* (https://mycocosm.jgi.doe.gov/Obbri1/Obbri1.home.html, accessed 10 July 2015) and *G. subvermispora* (https://mycocosm.jgi.doe.gov/Cersu1/Cersu1.home.html, accessed 10 July 2015) containing 13,206 and 12,125 predicted protein sequences, respectively, for peptide/protein identification as previously reported [23]. Protein area values were calculated using the top five most abundant identified peptides for each protein determined using the precursor ion quantitation workflow from Proteome Discoverer 2.4 (Thermo-Fisher) and were used to quantify relative levels of total proteins. Protein area values were normalized using the determined value for the spiked trypsin-digested bovine serum albumin internal standard. For each protein, mean abundance was calculated from two biological replicates and expressed as ion profile (IP) value. Proteins with IP value < 5 were filtered out from the analysis. The proteomics data have been deposited to the ProteomeXchange Consortium via the PRIDE partner repository with the dataset identifier PXD030609 and 10.6019/PDX030609. Principal component analysis (PCA) of the protein abundances on duplicate samples of the three-time points in two fungal species was performed with FactoMineR v1.7 package from R commander v.2.1-7 program (J. Fox, CRAN mirror in Utrecht, The Netherlands) in R statistical language and environment 3.1.2 [24]. Orthologous groups (OGs) were identified by utilizing OrthoMCL method [25] with parameters set as follows: E-value 1 × 10^−5^, inflation level 1.5, and sequence coverage 60%.

### 2.3. FTIR Analysis of Spruce Wood

The pooled samples from duplicate cultivations were analyzed by diffuse reflectance FTIR spectroscopy using a Bruker IFS 66V/S instrument (Bruker Optik GmbH, Ettlingen, Germany) under vacuum (4 mbar) conditions, according to the protocol by Gorzsás and Sundberg (2014) [26]: dried samples (1–10 mg) were mixed with infrared spectroscopy grade potassium bromide (KBr, ca. 390 mg) and manually ground using an agate mortar and pestle to obtain a homogeneous mixture. Spectra were recorded in the range of 400-4000 cm^−1^ at 4 cm^−1^ spectral resolution with 128 scans co-added, using pure KBr as background with the same parameters. Spectra were processed using the free, open source, Matlab-based script available from the Vibrational Spectroscopy Core Facility at Umeå University (www.umu.se/en/research/infrastructure/visp/downloads/, accessed on 11 April 2019) [27], using the following steps with the listed parameters: (1) Spectral region cut to 750–1850 cm^−1^ (fingerprint region); (2) baseline correction using asymmetrical least squares (lambda = 10,000,000, *p* = 0.001 (minimum); (3) total area normalisation over the cut spectral range; and (4) Savitzky–Golay smoothing (first order polynomial with a frame of three). No derivatisation or any other processing was applied after these steps.

## 3. Results and Discussion

### 3.1. Temporal Protein Profiles of O. rivulosa and G. subvermispora during Cultivation on Spruce

Proteomics analysis using LC-MS/MS was performed to investigate the lignocellulolytic protein profiles of *O. rivulosa* and *G. subvermispora* during growth on spruce wood sticks for 2, 4, and 8 weeks. The total number of detected proteins from the cultivations of *O. rivulosa* and *G. subvermispora* was 947 and 845, respectively (Supplementary Table S1c,f). Signal peptide was predicted for 14% of the proteins in both species, which is in line with the secreted proteins (15%) detected in white rot species *Phlebia radiata* cultivated on solid spruce wood [28]. Protein abundances from the biological replicate cultivations showed high coherence according to PCA analysis (Supplementary Figure S2). The genomes of both species used in this study harbor a broad set of predicted CAZymes targeted to degrade all polymeric components of wood cell walls. Their gene counts for CAZy family AA2 lignin modifying peroxidases and hydrogen peroxide producing family AA5_1 enzymes are typical for white rot species in Polyporales, while low numbers of laccase family AA1 and lytic polysaccharide monooxygenase (LPMO) family AA9 encoding genes resembled brown rot species in a sister clade Antrodia [29]. Of the detected proteins, 46 and 49 represented putative plant cell wall degrading CAZymes accounting for approximately 5% of the total proteome for both *O. rivulosa* and *G. subvermispora*, respectively (Supplementary Table S1a,d). Slightly higher proportion of plant cell wall degrading CAZymes (7%) has been detected in the total proteome of the white rot species Phlebia radiata cultivated on spruce wood [28]. Plant cell wall targeted CAZymes were classified into functional categories based on the substrate(s) on which they (putatively) are active.

Orthologous plant cell wall degrading CAZymes among *O. rivulosa* and *G. subvermispora* were identified and pairwise orthology relationship between them was inferred. In total, 64 common orthologous groups, a cluster of homologous sequences that diverged from the same speciation event, were found (Supplementary Table S1i,j). The analysis revealed the presence of orthologs in both proteomes for the majority of PCWDEs, indicating that *O. rivulosa* and *G. subvermispora* share a core set of orthologous PCWDEs during wood decay. In contrast, comparison of white and brown rot basidiomycete transcriptomes from various lignocellulose substrates has shown that only a few CAZyme orthologous gene groups are consistently upregulated that indicates diversity in their approaches to degrade plant biomass [30]. In *O. rivulosa* proteome, 80% of the plant cell wall degrading orthologs also were detected in *G. subvermispora*, while of the CAZy proteins produced by *G. subvermispora*, 15% were unique, and their orthologs were not produced by *O. rivulosa* (Supplementary Table S1i,j). Highly produced orthologs, which were not detected in *G. subvermispora*, were *O. rivulosa* LAC (GH35) and EGL (GH131) found at all time points, as well as GH27 (α-galactosidase, AGL), GH51 (α-arabinofuranosidase, ABF) and GH53 (β-endogalactanase, GAL), which were produced in 2-week cultivation of *O. rivulosa* (Supplementary Table S1i). *O. rivulosa* GH6 (CBHII) was produced in 2- and 4-week cultivations, whereas no CBHII was detected in *G. subvermispora* cultivations. In *G. subvermispora*, two H_2_O_2_-generating oxidases in CAZy families AA3_2 (aryl alcohol oxidase, AAO) and AA3_3 (alcohol oxidase, AOX) GH131 (EGL), a xyloglucan-acting GH31 (α-xylosidase, AXL) and CE1 (acetyl xylan esterase, AXE) were produced, but corresponding orthologs were not found in *O. rivulosa* (Supplementary Table S1j).

**Figure 1:**
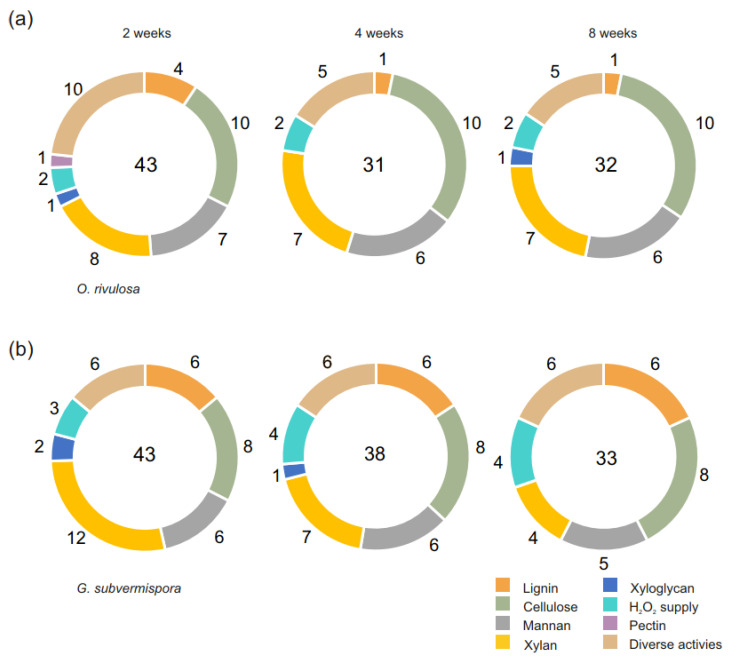
Functional distribution of detected predicted plant cell wall degrading CAZymes in spruce wood cultures of (**a**) *O. rivulosa* and (**b**) *G. subvermispora* on week 2, 4, and 8. Numbers in the center of the circles show the sum of detected plant cell wall degrading CAZymes in the specific time point. Numbers outside the circles display detected PCWDEs acting on their (putative) substrates at each time point.

A decrease in the number of PCWDEs, from 43 to 32, was detected between the 2- and 8-week spruce cultivations of *O. rivulosa* (Figure 1a). A similar trend was detected in the transcriptome of *O. rivulosa* when it was cultivated on spruce wood for 2, 4, and 8 weeks [31]. The highest number of PCWDEs (43) was detected in the 2-week cultures of *G. subvermispora*, with a clear decreasing trend after 4 and 8 weeks to 38 and 33 CAZymes, respectively (Figure 1b).

**Figure 2:**
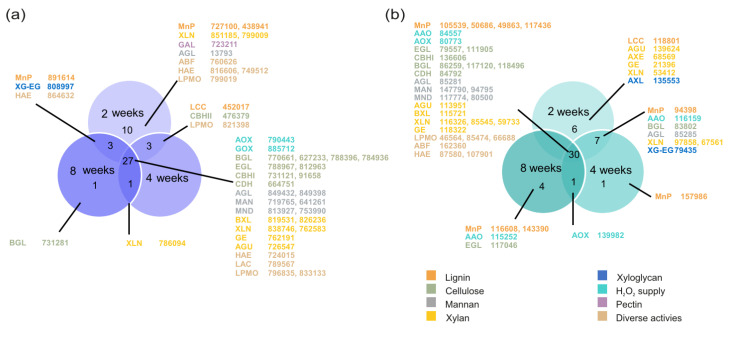
Venn diagrams showing the unique and common plant cell wall acting CAZymes in 2-, 4-, and 8-week solid spruce wood cultivations of (**a**) *O. rivulosa* and (**b**) *G. subvermispora*. CAZyme abbreviations (see Supplementary Table S1a,d) followed by JGI Protein IDs are depicted, and their font color is according to the substrate they are putatively acting on.

More than half of the total number of detected plant cell wall degrading CAZymes, 27 and 30, were produced in all studied time points in the cultivations of *O. rivulosa* and *G. subvermispora*, respectively (Figure 2, Supplementary Table S1g,h). In both species, most of these common proteins were cellulases and hemicellulases from diverse GH families. Lignin modification-related enzymes were common to all time points comprised of AA3 H_2_O_2_-supplying enzymes in both species, as well as AA2 manganese peroxidases (MnPs) in *G. subvermispora*. After 2-week cultivation, 11 and 6 CAZymes were exclusive for *O. rivulosa* and *G. subvermispora* cultures, respectively (Figure 2). The number of the unique proteins strongly decreased in the later time points of both fungi. None were detected in 4-week cultivations, and only one protein was present in 8-week cultivations of *O. rivulosa*. In *G. subvermispora* cultivation, one and four unique proteins were detected in 4- and 8-week cultivations, respectively (Figure 2b). In addition, one *O. rivulosa* and *G. subvermispora* PCWDE was shared between 4- and 8-week cultivations. Only *O. rivulosa* had PCWDEs that were shared between 2- and 8-week cultivations (Figure 2a).

### 3.2. Early Production of MnPs Suggests Their Important Role during the Beginning of Wood Decay

The number of lignin acting enzymes produced by *O. rivulosa* decreased during the cultivation, whereas in *G. subvermispora* the number of detected proteins were constant in all the time points during the 8-week cultivations (Figure 1). Three MnPs (Protein IDs: 727100, 891614, and 438941) out of 11 MnP encoding genes of *O. rivulosa* (https://genome.jgi.doe.gov/pages/search-for-genes.jsf?organism = Obbri1, accessed 5 April 2022) were produced in 2-week cultivations (Figure 2), which correlates well with our previous transcriptomic study where two MnPs (Prot. IDs: 727100 and 891614) were among the highest expressed PCWDE encoding genes during *O. rivulosa* cultivation on spruce [31]. *O. rivulosa* MnP 891614 was the only MnP among the most abundant PCWDEs produced (Table 1). Its production was the highest in 2-week cultivations, and only low amounts were produced at the later stages of cultivation. In *G. subvermispora* cultivations, MnPs were the most abundant lignin modifying enzymes produced with five detected isoenzymes (Protein IDs: 105539, 94398, 50686, 49863, and 117436) out of 13 MnP encoding genes (Table 2) [11]. Three highly produced MnPs showed a decreasing trend over time, similarly with a majority of the ten detected MnPs in the secretome of *G. subvermispora* (strain RP-B) grown in the ball-milled aspen containing liquid cultures, thus highlighting the importance of these enzymes in the early stages of wood degradation [32]. The most highly produced was MnP7 (Protein ID: 105539), which was detected throughout the cultivation, with an exceptionally high abundance in 8-week cultivations (Table 2). The amount of MnP10 (Protein ID: 117436) slightly increased during cultivation. Previously, MnP10 had been reported to be the most strongly upregulated MnP in ball-milled aspen containing liquid cultivations of *G. subvermispora* during seven days of cultivation [32]. As significant lignin loss in solid spruce wood has been detected after three days of *G. subvermispora* cultivation, selective lignin degradation is pronounced in very early stages of wood decay [33].

**Figure 3:**
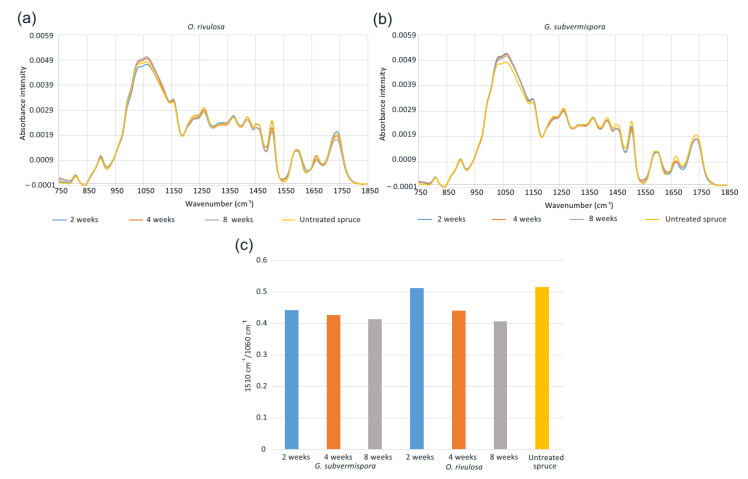
Diffuse reflectance FTIR spectra of Norway spruce wood samples after 2, 4, and 8 weeks of fungal cultivation: (**a**) FTIR spectra after *O. rivulosa* cultivation, (**b**) FTIR spectra after *G. subvermispora* cultivation, and (**c**) lignin (1510 cm^−1^)/polysaccharide (1060 cm^−1^) ratio.

This is supported by the spruce wood composition analyses after fungal growth by FTIR (Figure 3). Relative lignin/carbohydrate ratio was determined as the intensities of 1510 cm^−1^/1060 cm^−1^ [34] indicated loss in lignin in the early stages of *G. subvermispora* and *O. rivulosa* cultivation on spruce. *G. subvermispora* is more efficient than *O. rivulosa* in lignin degradation after two weeks of cultivation, yet the ratio is similar for both species after eight weeks. Similarly, degradation of lignin and hemicelluloses by *G. subvermispora* has been detected on oak wood during the first five weeks of cultivation [35].

In addition to MnPs, *O. rivulosa* and *G. subvermispora* encode one and two lignin peroxidase-type enzymes, respectively [14,18]. However, none were produced during 8-week cultivation on spruce. Detection of MnPs as the only lignin modifying peroxidases in *O. rivulosa* and *G. subvermispora* cultivations emphasized their importance in degradation of solid spruce wood. This is consistent with the previous studies on the production of MnPs as the main lignin modifying enzymes of *O. rivulosa* when grown on spruce wood chips [36,37]. Likewise, *G. subvermispora* (strain SS-3) has been shown to produce MnP as its main lignin modifying enzyme on loblolly pine and eucalyptus wood chips [38,39].

Besides lignin modifying peroxidases, laccases also are implicated in lignin degradation. Oxidation of wood lignin structures has been detected by fungal laccases either alone or in the presence of small molecular weight redox mediators [40,41,42]. Eight laccases are present in the genome of *O. rivulosa* [14], while *G. subvermispora* has seven predicted laccase encoding genes [11]. Unlike multiple MnP isoenzymes detected in the studied spruce stick cultures, only a single laccase isoenzyme was produced by *O. rivulosa* (Protein ID: 452017) after 2 and 4 weeks, and the corresponding laccase ortholog of *G. subvermispora* (Protein ID: 118801) was produced after 2 weeks. In addition, this laccase isoenzyme was the only lignin modifying enzyme produced by *G. subvermispora* after 2-week cultivation (Figure 2b). Low abundances and secretion limited to the early cultivation stage indicate that laccases may have restricted influence on lignin degradation in *O. rivulosa* and *G. subvermispora*, but they may have a role in initial wood colonization in natural conditions. Correspondingly, we have detected low and early expression of laccase-encoding genes in *O. rivulosa* spruce stick cultivations [31].

In the lignin degradation process by white rot fungi, an array of redox enzymes, such as family AA3 glucose–methanol–choline (GMC) oxidoreductases and AA5 copper radical oxidases (CROs), supplies H_2_O_2_ to class II peroxidases [43] as well as to LPMOs [44]. H_2_O_2_-supplying enzymes represented a small proportion of the plant cell wall degrading CAZymes in *O. rivulosa* (4%), whereas in *G. subvermispora* they constituted a notably higher fraction making 10% of detected PCWDEs. H_2_O_2_-generating family AA3_3 AOX enzymes were the most highly produced PCWDEs in *O. rivulosa* and *G. subvermispora* spruce cultures, thus indicating a very potent ligninolytic system in both species studied (Table 1 and Table 2). The highly produced *G. subvermispora* AOX (Protein ID: 80773) also has been abundantly produced in solid-state fermentation (SSF) of Jerusalem artichoke, together with an AA3_2 aryl alcohol oxidase (AAO) (Protein ID: 117387) [45]. Interestingly, this AAO has been the main H_2_O_2_-supplying enzyme in the secretome of *G. subvermispora* liquid ball-milled aspen cultures [32], but it was not detected in our study.

### 3.3. O. rivulosa and G. subvermispora Showed Constant Production of Cellulolytic Enzymes

Cellulose depolymerizing enzymes comprised the largest proportion of plant cell wall degrading CAZymes produced by *O. rivulosa* (23%) (Supplementary Table S1a). The number of cellulolytic enzymes stayed constant in both *O. rivulosa* and *G. subvermispora* cultures during 8-week cultivation (Figure 1).

A full enzymatic repertoire necessary for cellulose hydrolysis was produced by both species studied. The most highly produced cellulolytic enzymes in both species were GH7 cellobiohydrolase I (CBHI) targeted toward degradation of crystalline cellulose (Table 1 and Table 2). Interestingly, *O. rivulosa* and *G. subvermispora* produced the highest levels of CBHIs and other cellulases after 2 weeks, similar to lignin modifying enzymes, disputing the hypothesized selective lignin degradation approach in these conditions. Previously, minor depolymerization of cellulose had been detected in *G. subvermispora* (strain SS-3) eucalyptus wood chip cultures already 15 days of cultivation [46], which supports our finding of an active cellulolytic capacity. A repertoire of cellulose hydrolyzing enzymes was complemented by EGLs and BGLs, as well as oxidative LPMOs cleaving cellulose and other plant cell wall polysaccharides, and a cellobiose dehydrogenase (CDH) that serves as an external electron donor in LPMO-catalyzed reactions [47,48] (Table 1), which were present in both fungal proteomes grown on spruce wood. Both *O. rivulosa* and *G. subvermispora* with eight and nine putative AA9 LPMO encoding genes, respectively, indicate oxidative capacity for the degradation of plant polysaccharides. Two *O. rivulosa* LPMOs were among the most abundantly produced proteins in the spruce cultures, which is in line with the high transcript levels of their corresponding genes under similar cultivations [31]. All three *G. subvermispora* LPMOs detected from spruce cultivations in this study also have been upregulated at the transcript level on liquid ball-milled aspen medium; but surprisingly, none of those have been detected as proteins from liquid ball-milled aspen, Avicel or glucose cultivations [11]. Levels and production trends of CDH were in accordance with those of the LPMOs in both studied species. This indicates a certain discrepancy between LPMO expression and production levels. Inconsistencies between transcriptome and proteome data in fungal cultures have been reported, and they have been suggested to result from differential transcription and translation rate, mRNA, and protein stability as well as biochemical properties of proteins [21,49,50].

### 3.4. Early Production of Hemicellulases Coincided with Lignin Modifying Enzymes 

Xylanases accounted for the majority (28%) of the detected PCWDEs in *G. subvermispora* spruce cultivations (Figure 1b), while a smaller portion was observed in *O. rivulosa* cultivations (18%). Family GH5_7 mannan hydrolyzing β-1,4-endomannanase (MAN) (Protein ID: 641261) was the most abundant hemicellulase in the *O. rivulosa* spruce cultivations, indicating that this fungus has a good ability to convert spruce that has mannan as the main hemicellulose polymer [51]. We previously showed that GH5_7 MAN and GH10 β–1,4-endoxylanase (XLN) encoding genes were expressed at comparably high levels in the *O. rivulosa* spruce cultures after two weeks [31]. Although xylan is present in lower amounts in spruce (12.4 mol%) compared to mannan (16.7 mol%) [51], GH10 XLNs, which hydrolyzes β-1,4-bonds in the xylan backbone, were the main hydrolytic enzymes produced by *G. subvermispora* with all its six XLN isoenzymes detected. The two most highly produced XLNs on spruce wood (Protein IDs: 59733, 97858) also were detected from the ball-milled aspen liquid cultivations of *G. subvermispora* [32], whereas two different isoenzymes (Protein IDs: 67561, 116326) were identified in the exo-proteome of *G. subvermispora* from Jerusalem artichoke SSF cultivations [45], implying induction of different isoenzymes by different plant biomass substrates.

A relatively low presence of xyloglucanases, representing approximately 5% of the detected PCWDEs, was detected in both species. This reflects the low content of xyloglucan in softwood (10% (*w/w*) in the primary cell walls) [52]. Most of the hemicellulases in both species showed decreasing levels over the cultivation time, similarly as lignin modifying enzymes, thus supporting a simultaneous lignin degrading approach rather than a predicted selective lignin removal. The exceptions were two detected GH2 MNDs and two intracellular GH5_22 BXLs in *O. rivulosa* cultivations, and two GH10 XLNs and one CE15 glucuronoyl esterase (GE) in *G. subvermispora* cultivations, all of which were most abundant after 8 weeks (Supplementary Table S1d).

Diverse activities comprising enzymes, such as GH51 ABFs, CE16 hemicellulose acetyl esterases (HAEs), GH35 β-galactosidases (LACs), and AA9 LPMOs, putatively acting on several substrates, constituted 22% of all detected CAZymes in *O. rivulosa* and 18% in *G. subvermispora* (Supplementary Table S1b,e). The number of CAZymes with diverse activities decreased in the cultures of *O. rivulosa*, while in *G. subvermispora* the number stayed constant during the whole cultivation period (Figure 1). Interestingly, HAE was one of the most abundantly produced enzymes in *O. rivulosa*, while in *G. subvermispora* LPMOs were the most abundant diverse activities comprising enzymes.

GH51 ABFs act on side-chains of different arabinose-containing polysaccharides, such as xyloglucans, arabinoxylans, and pectin [53]. Although arabinose is only a minor constituent in wood accounting for 2.9 mol% in spruce [51], interestingly, one ABF isoenzyme was detected in the spruce cultivations of both species. The same *G. subvermispora* ABF (Protein ID: 162360) has been detected from SSF cultivation on Jerusalem artichoke that has higher arabinose content (5%) [45] as well as from the submerged ball-milled aspen cultures at the initial cultivation stage [32]. ABF has been speculated to indirectly contribute to lignin degradation; [32] although, there is no evidence of the existence of ferulic acid-linked arabinose in wood, which could be cross-linked to lignin.

## 4. Conclusions

In conclusion, both *O. rivulosa* and *G. subvermispora* produced all the necessary lignocellulolytic enzymes for the white rot type of decay of solid spruce wood, which involved an array of secreted cellulases, hemicellulases, lignin modifying enzymes, and auxiliary oxidoreductases. A core set of orthologous PCWDEs was shared suggesting similar approach for plant biomass degradation. Dynamics of the detected protein levels during the 8-week cultivation revealed that several lignin modifying class II peroxidases together with other oxidoreductases were abundantly produced after 2-week cultivation, suggesting an oxidative attack on lignin during the early growth stage on wood. Similar trends in abundances of class II peroxidases have previously been noticed for *O. rivulosa* [36], *G. subvermispora* [45], and other white rot species grown on solid lignocellulose substrates, such as Phlebia radiata [28].

Although selective lignin degradation has been postulated for *O. rivulosa* and *G. subvermispora*, in the conditions used in this study the obtained results did not corroborate these monokaryotic strains as selective lignin degraders. While enzymatic mechanisms behind selective lignin degradation remain still largely unsolved, it is known that this strategy can be affected by multiple parameters. One of the most prominent factors is temporal dependency, which limits lignin removal to an early decay stage, whereas hemicellulose and cellulose degradation appear later [8,54]. In addition, just like various strains of a species are known to show considerable variations in lignin selectivity, there are differences in degradation pattern between dikaryotic and monokaryotic strains of the same species [55,56,57]. Higher mycelial growth rate, enzyme activity levels, and broader induction of CAZyme encoding genes of dikaryotic strains compared to the monokaryotic strains, indicate an overall better ability to grow on and utilize plant biomass. Monokaryotic *O. rivulosa* and *G. subvermispora* showed that even though the majority of CAZymes that they produced during growth on spruce were orthologs, their diversity with respect to time-dependent production of plant cell wall degrading enzymes suggests a different strategy for spruce degradation for these taxonomically related species.

## Figures and Tables

**Table 1:** The 25 most abundant plant cell wall degrading CAZymes produced by *O. rivulosa* during growth on solid spruce wood. IP values = ion profile values. Protein IDs assigned by JGI MycoCosm (https://mycocosm.jgi.doe.gov/Obbri1/Obbri1.home.html, accessed 5 April 2022). Enzyme abbreviations are presented in Supplementary Table S1a.

				Protein Abundance (IP Values)		
				Time Point (Weeks)		
Protein ID	CAZy	Enzyme	Substrate/ Function	2	4	8
790443	AA3_3	AOX	H_2_O_2_-supply	408	132	144
641261	CBM1-GH5_7	MAN	galacto(gluco) mannan	214	68	70
838746	CBM1-GH10	XLN	xylan	166	26	14
724015	CE16	HAE	diverse	110	50	32
731121	GH7-CBM1	CBHI	cellulose	83	29	11
891614	AA2	MnP *	lignin	59	--	5
833133	AA9-CBM1	LPMO	diverse	50	22	27
821398	AA9	LPMO	diverse	46	15	--
812963	GH131	EGL	cellulose	43	16	15
851185	CBM1-GH10	XLN	xylan	43	--	--
789567	GH35	LAC	diverse	37	29	52
476379	CBM1-GH6	CBHII	cellulose	37	6	--
719765	GH5_7	MAN	galacto(gluco) mannan	36	17	19
885712	AA3_2	GOX	H_2_O_2_-supply	30	10	10
788967	CBM1-GH5_5	EGL	cellulose	28	13	24
849432	GH27	AGL	galacto(gluco) mannan	26	18	19
753990	GH2	MND	galacto(gluco) mannan	25	18	30
749512	CBM1-CE16	HAE	diverse	22	--	--
819531	GH5_22	BXL	xylan	21	32	48
813927	GH2	MND	galacto(gluco) mannan	21	14	29
664751	AA8-AA3_1	CDH	cellulose	21	6	7
816606	CE16	HAE	diverse	17	--	--
762191	CE15	GE	xylan	16	13	12
784936	GH3	BGL	cellulose	15	13	13
627233	GH3	BGL	cellulose	15	10	22

* Hakala et al. (2006) [36].

**Table 2:** The 25 most abundant plant cell wall degrading CAZymes produced by *G. subvermispora* during growth on solid spruce wood. IP values = ion profile values. Protein IDs assigned by JGI MycoCosm (https://mycocosm.jgi.doe.gov/Cersu1/Cersu1.home.html, accessed 5 April 2022). Enzyme abbreviations are presented in Supplementary Table S1d.

				Protein Abundance (IP Values)		
				Time Point (Weeks)		
Protein_ID	CAZy	Enzyme	Substrate/ Function	2	4	8
80773	AA3_3	AOX	H_2_O_2_-supply	739	163	266
105539	AA2	MnP	lignin	263	242	1218
59733	CBM1-GH10	XLN	xylan	176	60	19
49863	AA2	MnP	lignin	133	40	12
94795	CBM1-GH5_7	MAN	galacto(gluco) mannan	110	61	9
46564	AA9	LPMO	diverse	92	59	31
136606	GH7-CBM1	CBHI	cellulose	85	68	5
115721	GH5_22	BXL	xylan	77	40	36
66688	AA9-CBM1	LPMO	diverse	73	72	8
50686	AA2	MnP	lignin	56	146	16
80500	GH2	MND	galacto(gluco) mannan	46	21	18
97858	CBM1-GH10	XLN	xylan	43	16	--
84557	AA3_2	AAO	H_2_O_2_-supply	42	38	47
84792	AA8-AA3_1	CDH	cellulose	39	26	12
162360	GH51	ABF	diverse	34	15	9
94398	AA2	MnP	lignin	26	4	--
116159	AA3_2	AAO	H_2_O_2_-supply	25	6	--
118322	CE15	GE	xylan	24	18	39
147790	GH5_7	MAN	galacto(gluco) mannan	23	15	14
118801	AA1_1	LCC	lignin	23	--	--
85281	GH27	AGL	galacto(gluco) mannan	22	13	16
117120	GH3	BGL	cellulose	22	10	8
117436	AA2	MnP	lignin	21	23	36
67561	CBM1-GH10	XLN	xylan	20	5	--
79557	CBM1-GH5_5	EGL	cellulose	18	16	6

## Data Availability

The proteomics data have been deposited to the ProteomeXchange Consortium via the PRIDE partner repository (www.ebi.ac.uk/pride/) and are accessible with the data set identifier PXD030609 and 10.6019/PDX030609.

## Supplementary Materials

The following supporting information can be downloaded at: https://www.mdpi.com/article/10.3390/biom12081017/s1, Figure S1: Fungal growth on spruce wood sticks for 2, 4, and 8 weeks. (a) *O. rivulosa* monokaryon 3A-2 and (b) *G. subvermispora* monokaryon RP-B; Figure S2: Principal component analysis (PCA) of the proteomes of (a) *O. rivulosa* and (b) *G. subvermispora*. Table S1: Plant cell wall degrading CAZymes and their respective orthologs detected in the proteome of *O. rivulosa* and *G. subvermispora*.

## References

[1] Mäkelä M.R., Hildén K.S., de Vries R.P., Nowrousian M. (2014). Degradation and modification of plant biomass by fungi. Fungal Genomics. The Mycota.

[2] Lundell T., Mäkelä M.R., de Vries R.P., Hildén K.S., Martin F. (2014). Genomics, life-styles and future prospects of wood-decaying and litter-decomposing Basidiomycota. Advances in Botanical Research.

[3] Hatakka A., Hammel K.E., Hofrichter M. (2011). Fungal biodegradation of lignocelluloses. Industrial Applications, The Mycota.

[4] Lombard V., Golaconda Ramulu H., Drula E., Coutinho P.M., Henrissat B. (2014). The carbohydrate-active enzymes database (CAZy) in 2013. Nucleic Acids Res..

[5] Korripally P., Hunt C.G., Houtman C.J., Jones D.C., Kitin P.J., Cullen D., Hammel K.E. (2015). Regulation of gene expression during the onset of ligninolytic oxidation by *Phanerochaete chrysosporium* on spruce wood. Appl. Environ. Microbiol..

[6] Akhtar M., Blanchette R.A., Kirk T.K., Eriksson K.-E. (1997). Fungal delignification and biomechanical pulping of wood. Biotechnology in the Pulp and Paper Industry.

[7] Gupta R., Mehta G., Khasa Y.P., Kuhad R.C. (2011). Fungal delignification of lignocellulosic biomass improves the saccharification of cellulosics. Biodegradation.

[8] Hakala T.K., Maijala P., Konn J., Hatakka A. (2004). Evaluation of novel wood-rotting polypores and corticioid fungi for the decay and biopulping of Norway spruce (*Picea abies*) wood. Enz. Microb. Technol..

[9] Adaskaveg J.E., Gilbertson R.L., Dunlap M.R. (1995). Effects of incubation time and temperature on in vitro selective delignification of silver leaf oak by *Ganoderma colossum*. Appl. Environ. Microbiol..

[10] Blanchette R.A. (1995). Degradation of lignocellulose complex in wood. Can. J. Bot..

[11] Fernandez-Fueyo E., Ruiz-Dueñas F.J., Ferreira P., Floudas D., Hibbett D.S., Canessa P., Larrondo L.F., James T.Y., Seelenfreund D., Lobos S. (2012). Comparative genomics of *Ceriporiopsis subvermispora* and *Phanerochaete chrysosporium* provide insight into selective ligninolysis. Proc. Natl. Acad. Sci. USA.

[12] Binder M., Justo A., Riley R., Salamov A., Lopez-Giraldez F., Sjokvist E., Copeland A., Foster B., Sun H., Larsson E. (2013). Phylogenetic and phylogenomic overview of the Polyporales. Mycologia.

[13] Justo A., Miettinen O., Floudas D., Ortiz-Santana B., Sjokvist E., Lindner D., Nakasone K., Niemelä T., Larsson K.H., Ryvarden L. (2017). A revised family-level classification of the Polyporales (Basidiomycota). Fungal Biol..

[14] Miettinen O., Riley R., Barry K., Cullen D., de Vries R.P., Hainaut M., Hatakka A., Henrissat B., Hilden K., Kuo R. (2016). Draft genome sequence of the white-rot fungus *Obba rivulosa* 3A-2. Genome Announc..

[15] Sun Y.F., Lebreton A., Xing J.H., Fang Y.X., Si J., Morin E., Miyauchi S., Drula E., Ahrendt S., Cobaugh K. (2022). Phylogenomics and comparative genomics highlight specific genetic features in *Ganoderma* species. J. Fungi.

[16] Altenhoff A.M., Glover N.M., Dessimoz C. (2019). Inferring orthology and paralogy. Methods Mol. Biol..

[17] Benoit I., Culleton H., Zhou M., DiFalco M., Aguilar-Osorio G., Battaglia E., Bouzid O., Brouwer C., El-Bushari H.B.O., Coutinho P.M. (2015). Closely related fungi employ diverse enzymatic strategies to degrade plant biomass. Biotechnol. Biofuels.

[18] Fernandez-Fueyo E., Ruiz-Dueñas F.J., Miki Y., Martinez M.J., Hammel K.E., Martinez A.T. (2012). Lignin-degrading peroxidases from genome of selective ligninolytic fungus *Ceriporiopsis subvermispora*. J. Biol. Chem..

[19] Hatakka A.I., Uusi-Rauva A.K. (1983). Degradation of ^14^C-labelled poplar wood lignin by selected white-rot fungi. Eur. J. Appl. Microbiol. Biotechnol..

[20] Mäkelä M., Galkin S., Hatakka A., Lundell T. (2002). Production of organic acids and oxalate decarboxylase in lignin-degrading white rot fungi. Enzym. Microb. Technol..

[21] Rytioja J., Hilden K., Di Falco M., Zhou M., Aguilar-Pontes M.V., Sietio O.M., Tsang A., de Vries R.P., Mäkelä M.R. (2017). The molecular response of the white-rot fungus *Dichomitus squalens* to wood and non-woody biomass as examined by transcriptome and exoproteome analyses. Environ. Microbiol..

[22] Mahajan C., Basotra N., Singh S., Di Falco M., Tsang A., Chadha B.S. (2016). Malbranchea cinnamomea: A thermophilic fungal source of catalytically efficient lignocellulolytic glycosyl hydrolases and metal dependent enzymes. Bioresour. Technol..

[23] Budak S.O., Zhou M., Brouwer C., Wiebenga A., Benoit I., Di Falco M., Tsang A., de Vries R.P. (2014). A genomic survey of proteases in Aspergilli. BMC Genom..

[24] Lê S., Josse J., Husson F. (2008). FactoMineR: An R package for multivariate analysis. J. Stat. Softw..

[25] Li L., Stoeckert C.J., Roos D.S. (2003). OrthoMCL: Identification of ortholog groups for eukaryotic genomes. Genome Res..

[26] Gorzsás A., Sundberg B. (2014). Chemical fingerprinting of *Arabidopsis* using Fourier Transform Infrared (FT-IR) spectroscopic approaches. Methods Mol. Biol..

[27] Felten J., Hall H., Jaumot J., Tauler R., de Juan A., Gorzsás A. (2019). Addendum: Vibrational spectroscopic image analysis of biological material using multivariate curve resolution–alternating least squares (MCR-ALS). Nat. Protoc..

[28] Kuuskeri J., Häkkinen M., Laine P., Smolander O.P., Tamene F., Miettinen S., Nousiainen P., Kemell M., Auvinen P., Lundell T. (2016). Time-scale dynamics of proteome and transcriptome of the white-rot fungus *Phlebia radiata*: Growth on spruce wood and decay effect on lignocellulose. Biotechnol. Biofuels.

[29] Hage H., Miyauchi S., Virágh M., Drula E., Min B., Chaduli D., Navarro D., Favel A., Norest M., Lesage-Meessen L. (2021). Gene family expansions and transcriptome signatures uncover fungal adaptations to wood decay. Environ. Microbiol..

[30] Peng M., Aguilar-Pontes M.V., Hainaut M., Henrissat B., Hilden K., Mäkelä M.R., de Vries R.P. (2018). Comparative analysis of basidiomycete transcriptomes reveals a core set of expressed genes encoding plant biomass degrading enzymes. Fungal Genet. Biol..

[31] Marinovic M., Aguilar-Pontes M.V., Zhou M., Miettinen O., de Vries R.P., Mäkelä M.R., Hilden K. (2018). Temporal transcriptome analysis of the white-rot fungus *Obba rivulosa* shows expression of a constitutive set of plant cell wall degradation targeted genes during growth on solid spruce wood. Fungal Genet. Biol..

[32] Hori C., Gaskell J., Igarashi K., Kersten P., Mozuch M., Samejima M., Cullen D. (2014). Temporal alterations in the secretome of the selective ligninolytic fungus *Ceriporiopsis subvermispora* during growth on aspen wood reveal this organism’s strategy for degrading lignocellulose. Appl. Environ. Microbiol..

[33] Fackler K., Gradinger C., Schmutzer M., Tavzes C., Burgert I., Schwanninger M., Hinterstoisser B., Watanabe T., Messner K. (2007). Biotechnological wood modification with selective white-rot fungi and its molecular mechanisms. Food Technol. Biotechnol..

[34] Pânzariu A.E., Măluţan T., Mangalagiu I. (2014). The hydrolysis of cellulosic materials in ionic liquids. BioResources.

[35] Van Kuijk S.J.A., Sonnenberg A.S.M., Baars J.J.P., Hendriks W.H., del Río J.C., Rencoret J., Gutiérrez A., de Ruijter N.C.A., Cone J.W. (2017). Chemical changes and increased degradability of wheat straw and oak wood chips treated with the white rot fungi *Ceriporiopsis subvermispora* and *Lentinula edodes*. Biomass Bioenergy.

[36] Hakala T.K., Hilden K., Maijala P., Olsson C., Hatakka A. (2006). Differential regulation of manganese peroxidases and characterization of two variable MnP encoding genes in the white-rot fungus *Physisporinus rivulosus*. Appl. Microbiol. Biotechnol..

[37] Hakala T.K., Lundell T., Galkin S., Maijala P., Kalkkinen N., Hatakka A. (2005). Manganese peroxidases, laccases and oxalic acid from the selective white-rot fungus *Physisporinus rivulosus* grown on spruce wood chips. Enzym. Microb. Technol..

[38] Aguiar A., de Souza-Cruz P.B., Ferraz A. (2006). Oxalic acid, Fe^3+^-reduction activity and oxidative enzymes detected in culture extracts recovered from *Pinus taeda* wood chips biotreated by *Ceriporiopsis subvermispora*. Enzym. Microb. Technol..

[39] Vicentim M.P., Ferraz A. (2007). Enzyme production and chemical alterations of *Eucalyptus grandis* wood during biodegradation by *Ceriporiopsis subvermispora* in cultures supplemented with Mn^2+^, corn steep liquor and glucose. Enzym. Microb. Technol..

[40] Kontro J., Maltari R., Mikkilä J., Kähkönen M., Mäkelä M.R., Hildén K., Nousiainen P., Sipilä J. (2020). Applicability of recombinant laccases from the white-rot fungus *Obba rivulosa* for mediator-promoted oxidation of biorefinery lignin at low pH. Front. Bioeng. Biotechnol..

[41] Moilanen U., Kellock M., Várnai A., Andberg M., Viikari L. (2014). Mechanisms of laccase-mediator treatments improving the enzymatic hydrolysis of pre-treated spruce. Biotechnol. Biofuels.

[42] Munk L., Sitarz A.K., Kalyani D.C., Mikkelsen J.D., Meyer A.S. (2015). Can laccases catalyze bond cleavage in lignin?. Biotechnol. Adv..

[43] Mäkelä M.R., Hildén K.S., Kuuskeri J., Zaragoza Ó., Casadevall A. (2021). Fungal lignin-modifying peroxidases and H_2_O_2_-producing enzymes. Encyclopedia of Mycology.

[44] Bissaro B., Rohr A.K., Muller G., Chylenski P., Skaugen M., Forsberg Z., Horn S.J., Vaaje-Kolstad G., Eijsink V.G.H. (2017). Oxidative cleavage of polysaccharides by monocopper enzymes depends on H_2_O_2_. Nat. Chem. Biol..

[45] Zhu N., Liu J., Yang J., Lin Y., Yang Y., Ji L., Li M., Yuan H. (2016). Comparative analysis of the secretomes of *Schizophyllum commune* and other wood-decay basidiomycetes during solid-state fermentation reveals its unique lignocellulose-degrading enzyme system. Biotechnol. Biofuels.

[46] Ferraz A., Córdova A.M., Machuca A. (2003). Wood biodegradation and enzyme production by *Ceriporiopsis subvermispora* during solid-state fermentation of Eucalyptus grandis. Enzym. Microb. Technol..

[47] Langston J.A., Shaghasi T., Abbate E., Xu F., Vlasenko E., Sweeney M.D. (2011). Oxidoreductive cellulose depolymerization by the enzymes cellobiose dehydrogenase and glycoside hydrolase 61. Appl. Environ. Microbiol..

[48] Tan T.C., Kracher D., Gandini R., Sygmund C., Kittl R., Haltrich D., Hallberg B.M., Ludwig R., Divne C. (2015). Structural basis for cellobiose dehydrogenase action during oxidative cellulose degradation. Nat. Commun..

[49] Patyshakuliyeva A., Post H., Zhou M., Jurak E., Heck A.J., Hilden K.S., Kabel M.A., Mäkelä M.R., Altelaar M.A., de Vries R.P. (2015). Uncovering the abilities of *Agaricus bisporus* to degrade plant biomass throughout its life cycle. Environ. Microbiol..

[50] Vogel C., Marcotte E.M. (2012). Insights into the regulation of protein abundance from proteomic and transcriptomic analyses. Nat. Rev. Genet..

[51] Daly P., Lopez S.C., Peng M., Lancefield C.S., Purvine S.O., Kim Y.M., Zink E.M., Dohnalkova A., Singan V.R., Lipzen A. (2018). *Dichomitus squalens* partially tailors its molecular responses to the composition of solid wood. Environ. Microbiol..

[52] Scheller H.V., Ulvskov P. (2010). Hemicelluloses. Annu. Rev. Plant Biol..

[53] Rytioja J., Hilden K., Yuzon J., Hatakka A., de Vries R.P., Mäkelä M.R. (2014). Plant-polysaccharide-degrading enzymes from Basidiomycetes. Microbiol. Mol. Biol. Rev..

[54] Blanchette R.A. (1991). Delignification by wood-decay fungi. Annu. Rev. Phytopathol..

[55] Casado Lopez S., Peng M., Issak T.Y., Daly P., de Vries R.P., Mäkelä M.R. (2018). Induction of genes encoding plant cell wall-degrading carbohydrate-active enzymes by lignocellulose-derived monosaccharides and cellobiose in the white-rot fungus *Dichomitus squalens*. Appl. Environ. Microbiol..

[56] Casado Lopez S., Theelen B., Manserra S., Issak T.Y., Rytioja J., Mäkelä M.R., de Vries R.P. (2017). Functional diversity in *Dichomitus squalens* monokaryons. IMA Fungus.

[57] Liu T., Li H., Ding Y., Qi Y., Gao Y., Song A., Shen J., Qiu L. (2017). Genome-wide gene expression patterns in dikaryon of the basidiomycete fungus *Pleurotus ostreatus*. Braz. J. Microbiol..

