# Supplementary material for: Comparative Analysis of Enzyme Production Patterns of Lignocellulose Degradation of Two White Rot Fungi: Obba rivulosa and Gelatoporia subvermispora
Source: Biomolecules. 2022 Jul 22;12(8):1017. doi: 10.3390/biom12081017 (PMC9330253; doi:10.3390/biom12081017)
Supplement: Supplementary file 1 [file biomolecules-12-01017-s001.zip › biomolecules-1773565-supplementary/Figure S1.pdf]

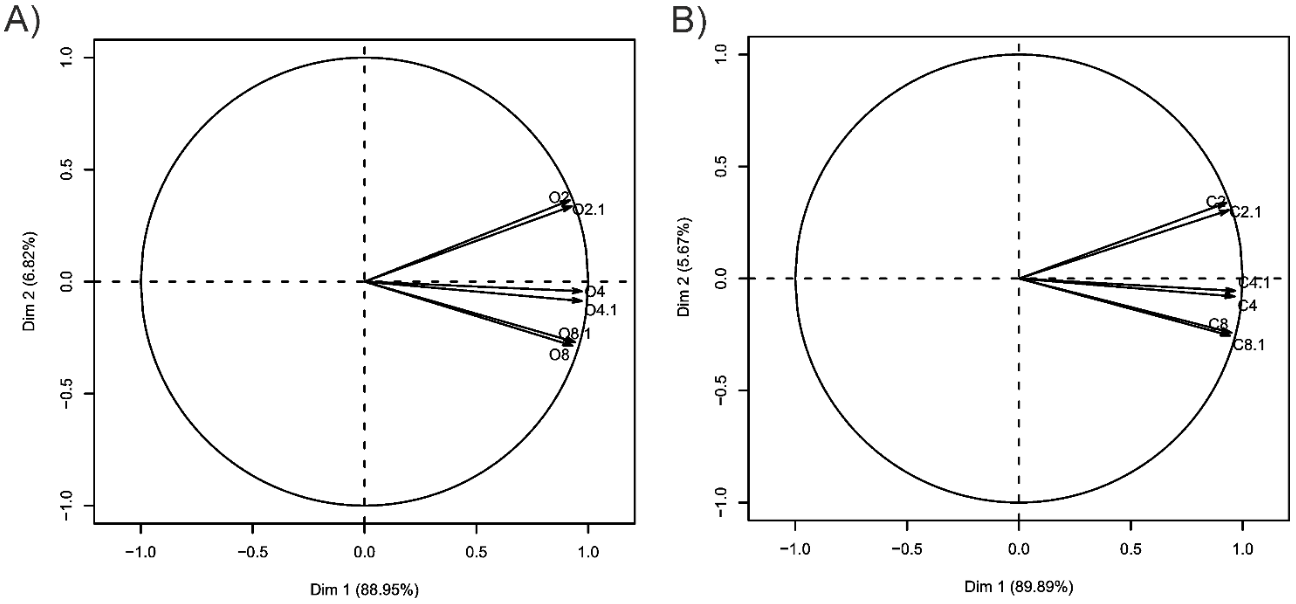


**Figure S1.** Principal component analysis (PCA) of the proteomes of (a) *O. rivulosa* and (b) *G. subvermispora*. Two biological replicates used for the LC-MS/MS analysis from 2-, 4- and 8-week cultivations on spruce wood are shown. O_2 and O_2.1, *O. rivulosa* 2-week samples; O_4 and O_4.1, *O. rivulosa* 4-week samples; O_8 and O_8.1, *O. rivulosa* 8-week samples. C_2 and C_2.1, *G. subvermispora* 2-week samples; C_4 and C_4.1, *G. subvermispora* 4-week samples; C_8 and C_8.1, *G. subvermispora* 8-week samples.
