# Supplementary material for: Comparative Analysis of Enzyme Production Patterns of Lignocellulose Degradation of Two White Rot Fungi: Obba rivulosa and Gelatoporia subvermispora
Source: Biomolecules. 2022 Jul 22;12(8):1017. doi: 10.3390/biom12081017 (PMC9330253; doi:10.3390/biom12081017)
Supplement: Supplementary file 1 [file biomolecules-12-01017-s001.zip › biomolecules-1773565-supplementary/Figure S2.pdf]

(a)

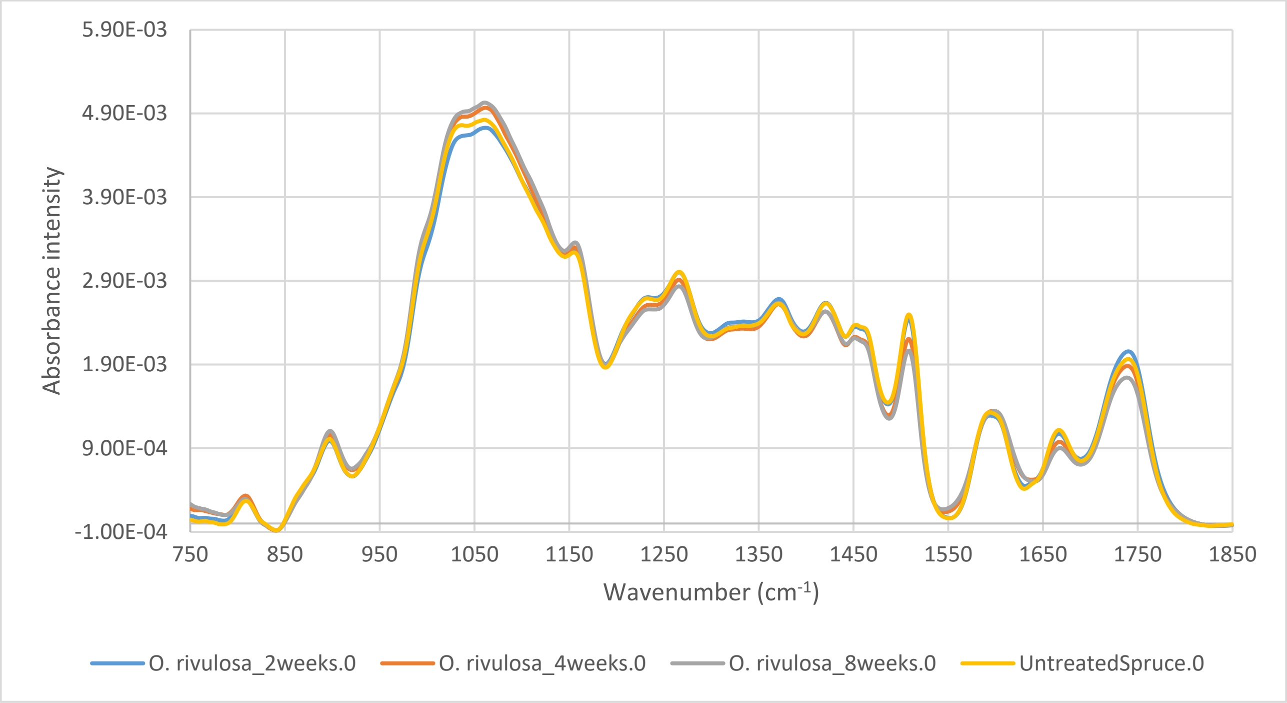

5.90E-03
4.90E-03
3.90E-03
2.90E-03
1.90E-03
9.00E-04
-1.00E-04
Absorbance intensity
750
850
950
1050
1150
1250
1350
1450
1550
1650
1750
1850
Wavenumber (cm-1)
O. rivulosa_2weeks.0
O. rivulosa_4weeks.0
O. rivulosa_8weeks.0
UntreatedSpruce.0

(b)

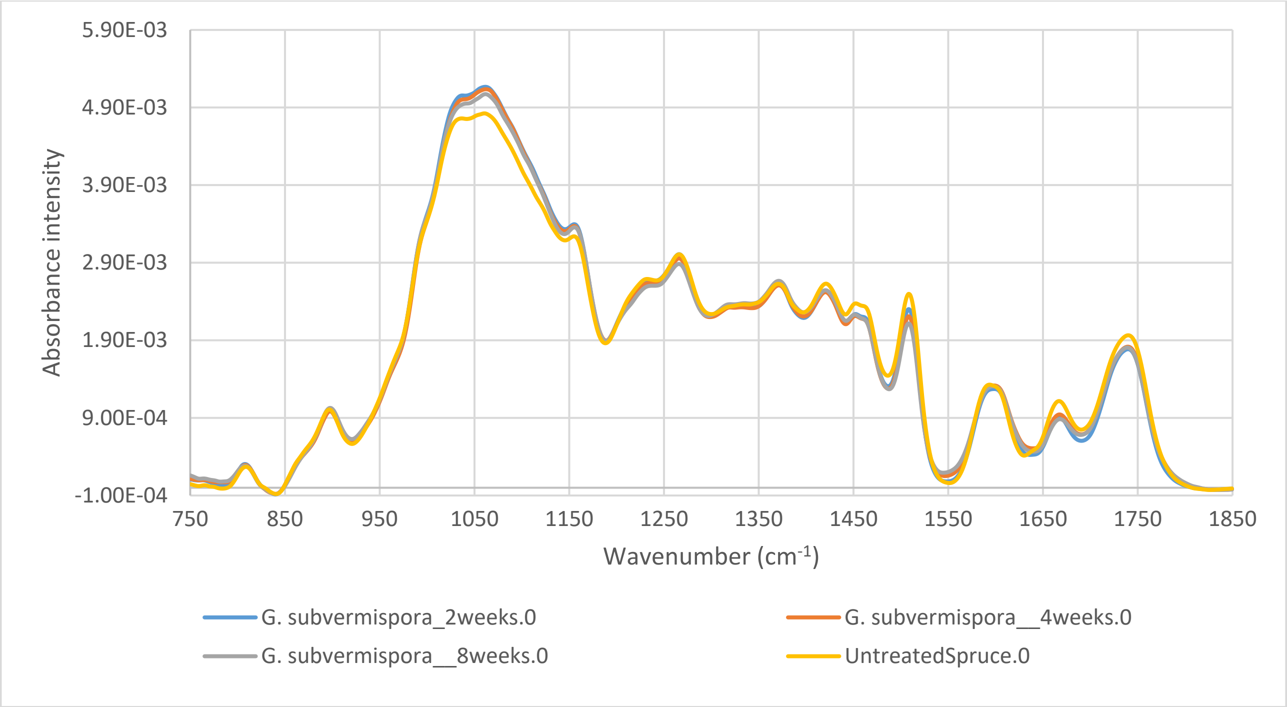

5.90E-03
4.90E-03
3.90E-03
2.90E-03
1.90E-03
9.00E-04
-1.00E-04
Absorbance intensity
750
850
950
1050
1150
1250
1350
1450
1550
1650
1750
1850
Wavenumber (cm-1)
G. subvermispora_2weeks.0
G. subvermispora__4weeks.0
G. subvermispora__8weeks.0
UntreatedSpruce.0

(c)

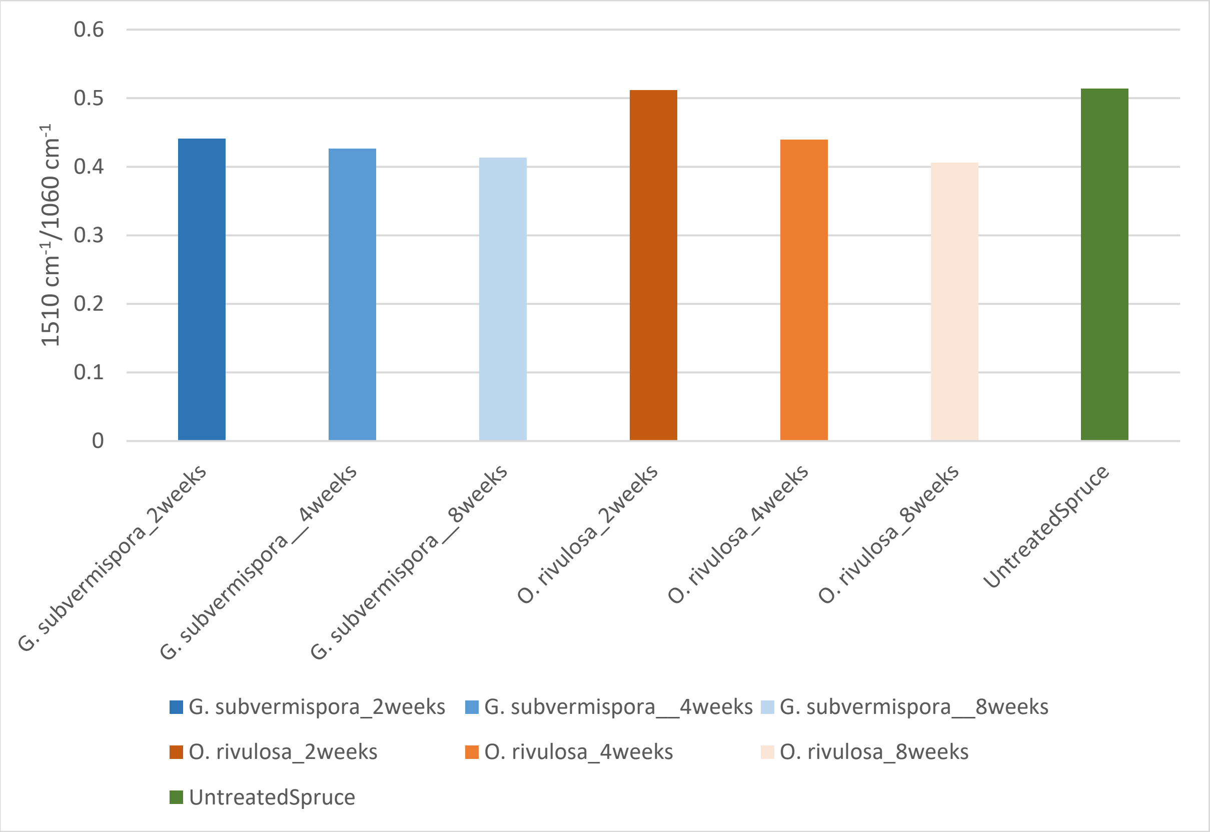

Figure S2. Diffuse reflectance FTIR spectra of Norway spruce wood samples after for 2, 4 and 8 weeks of fungal cultivation. (a) FTIR spectra after *O. rivulosa* cultivation. (b) FTIR spectra after *G. subvermispora* cultivation and (c) lignin (1510 cm^{-1})/polysaccharide (1060 cm^{-1}) ratio.
